# Effect of Water Addition on Gel-Forming Capacity and Structure–Property Evolution of Water-Reactive Polyurethane Grout

**DOI:** 10.3390/polym18212731

**Published:** 2026-11-03

**Authors:** Bowen Qi, Xinhua Chen, Langtian Qin, Feng Huang

**Affiliations:** School of Engineering and Technology, China University of Geosciences (Beijing), Beijing 100083, China; 1002255123@email.cugb.edu.cn (B.Q.); 2102260139@email.cugb.edu.cn (X.C.); 3002260044@email.cugb.edu.cn (L.Q.)

**Keywords:** water-reactive polyurethane, water addition, gelation behavior, mechanical properties, microstructure, molecular dynamics

## Abstract

Water-reactive polyurethane (PU) grout is widely used for water sealing, yet its gel-forming capacity and property evolution under uncontrolled water addition remain insufficiently understood. In this study, PU:water volume ratios from 1:1 to 1:20 were investigated using gelation tests, Shore OO hardness and uniaxial compression tests, SEM, ATR-FTIR, and non-reactive all-atom molecular dynamics simulations. Increasing water addition prolonged the gelation time from approximately 15 s at PU:water = 1:1 to 22 s at 1:15. Stable gel formation was maintained up to a water-addition multiple of 18× (PU:water = 1:18, *v*/*v*), whereas stable gel formation failed at multiples of 19× and 20×. Meanwhile, the Shore OO hardness decreased from approximately 63 to 2, and the compressive stress at 50% strain decreased from 208 to 8 kPa between PU:water = 1:1 and 1:15. SEM revealed increasingly heterogeneous and locally loose pore structures at high water contents. ATR-FTIR confirmed substantial consumption of free –NCO groups and changes in the carbonyl/amide region after curing. MD simulations further showed composition-dependent changes in local water distribution and hydrogen-bond participation before reaction. These results establish the water-tolerance boundary and multiscale property evolution of water-reactive PU, providing a basis for controlling PU–water proportions in water-rich grouting applications.

## 1. Introduction

In tunnel engineering [1], hydropower projects [2], mining [3], and urban underground space construction [4], water-rich and highly permeable strata can induce groundwater inflow, seepage erosion, and ground instability. Grouting is an important technique for water-hazard control and ground reinforcement because hardenable grout can be injected into pores and fractures to reduce permeability and improve the overall stability of the surrounding ground [5,6]. However, under water-rich conditions, particularly in flowing water, injected grout is susceptible to dilution and washout. If the grout cannot gel rapidly and form a stable consolidated body, its retention and sealing efficiency may be significantly reduced [7,8,9]. Therefore, grouting materials used under flowing-water conditions are generally required to exhibit a short gelation time together with good resistance to dilution and washout [10,11,12].

Water-reactive polyurethane (PU) grout is a representative chemical grouting material that can meet these requirements [12]. The isocyanate groups (–NCO) in such materials react with water, leading to CO_2_ generation and the subsequent formation of urea structures. The released CO_2_ promotes bubble nucleation and expansion, resulting in the gradual formation of a porous consolidated structure [13,14]. Meanwhile, hydrogen bonding involving urethane and urea groups plays an important role in determining the microstructure and macroscopic properties of PU materials [15]. Water is therefore not only the surrounding medium of water-reactive PU but also an active component directly involved in foaming, curing, and structural development. In practical grouting under water-rich conditions, the local mixing ratio between the grout and groundwater is difficult to control precisely. Clarifying the gelation behavior and final properties of PU under different levels of water addition is therefore important for evaluating its applicability to water-sealing applications.

Previous studies have demonstrated that water addition is an important factor affecting the curing reaction, pore structure, and mechanical properties of PU materials. Vipulanandan et al. [16] investigated hydrophilic PU grouts with different water-to-grout ratios and examined curing pressure, temperature, volume change, and compressive response, demonstrating that the water-to-grout ratio markedly influences both the curing process and the mechanical behavior of the consolidated grout. Vipulanandan et al. [17] subsequently extended the water-to-grout ratio to 0.5–8 and established relationships between the ratio and the maximum curing pressure, temperature rise, and volumetric expansion, showing that both the maximum pressure and temperature rise generally decreased with increasing water-to-grout ratio. In water-blown PU systems, Liang et al. [18] reported that increasing water addition enlarged the cellular size while reducing both tensile and compressive strengths. Chen et al. [19] further showed, using SEM and FTIR analyses, that changes in water content affected not only cellular morphology and mechanical properties but also the chemical structure of PU. Cimavilla-Román et al. [20] employed cryogenic X-ray micro-computed tomography to characterize the three-dimensional evolution of PU during the early stage of foaming and found that increasing water content had a relatively limited influence on the number of nucleated cells but accelerated cell growth and reduced the relative density of the system.

Related studies have also examined PU from the perspectives of cellular structure, chemical structure, hydrogen bonding, and molecular behavior. Hasan et al. [21] combined SEM and FTIR to investigate structural changes in PU foams prepared with different water contents and found that increasing water content promoted cell expansion, reduced cell-wall thickness, and altered chemical structural features associated with urea groups. Wang et al. [22] investigated the evolution of the chemical and microphase structures of PU during water absorption and found that water molecules preferentially interacted with free carbonyl groups before further affecting hydrogen-bonded carbonyl groups and the organization of hard-segment microdomains. At the molecular scale, Zhou and Choi [23] employed molecular dynamics (MD) simulations to investigate water diffusion in PU at different water contents and showed that PU–water hydrogen bonding and polymer swelling play important roles in controlling water transport. Varady et al. [24] further demonstrated that competitive hydrogen-bonding interactions among different components in PU can markedly affect molecular diffusion. Wu et al. [25] combined microstructural characterization with MD simulations to analyze the micromechanical response of foamed PU and related cellular features to the mechanical behavior of the material. Chen et al. [26] used MD simulations to investigate water permeation through PU nanochannels and revealed hydrogen bonding near polar PU groups, local aggregation of water molecules, and the evolution of water–water hydrogen-bond networks.

These studies provide an important basis for understanding the effects of water on the reaction, structure, and properties of PU materials. Nevertheless, during practical water-sealing grouting, the local mixing ratio between water-reactive PU and groundwater after injection is difficult to control accurately. Although the effects of water content on PU materials have been investigated previously, the upper limit of stable gel formation under high water addition and the associated evolution of mechanical properties, microstructure, and chemical structure have not been comprehensively examined together with the pre-reaction molecular environment. The novelty of this study lies in the combination of experimentally determining the upper limit of stable gel formation, systematically characterizing the associated multiscale structural and property changes, and using non-reactive molecular dynamics simulations to provide complementary insights into the pre-reaction PU–water environment. To this end, a water-reactive PU grouting material was investigated at PU:water volume ratios ranging from 1:1 to 1:20, with additional ratios introduced to determine the critical gel-forming range. Gelation behavior, mechanical properties, microstructure, and chemical structure were evaluated using gelation tests, Shore hardness and uniaxial compression tests, SEM, and ATR-FTIR, respectively. Non-reactive all-atom MD simulations were further employed to characterize the local distribution and hydrogen-bonding interactions of water in the pre-reaction PU–water systems without explicitly modeling chemical reactions. By integrating experimental characterization with molecular-level analysis, this study establishes the stable gel-forming limit under the investigated conditions and provides a comprehensive understanding of the effects of water addition on water-reactive PU grout, offering a basis for its application in water-rich grouting conditions.

## 2. Materials and Methods

### 2.1. Materials

YH-201 water-reactive polyurethane grouting material supplied by Beijing Oriental Yuhong Waterproof Technology Co., Ltd. (Beijing, China) was used in this study. The material is a pale-yellow liquid with relatively low viscosity at room temperature and can self-emulsify and rapidly form a gel upon contact with water. The isocyanate groups (–NCO) in the material react with water, accompanied by the generation of carbon dioxide, thereby promoting the formation of a porous polyurethane structure. Deionized water was used throughout the experiments to minimize the potential effects of dissolved ions and other impurities on the water–polyurethane reaction. Prior to testing, the polyurethane grouting material and deionized water were allowed to equilibrate at room temperature, and all experiments were conducted under ambient laboratory conditions.

### 2.2. Specimen Preparation and Mix Design

**Figure 1:**
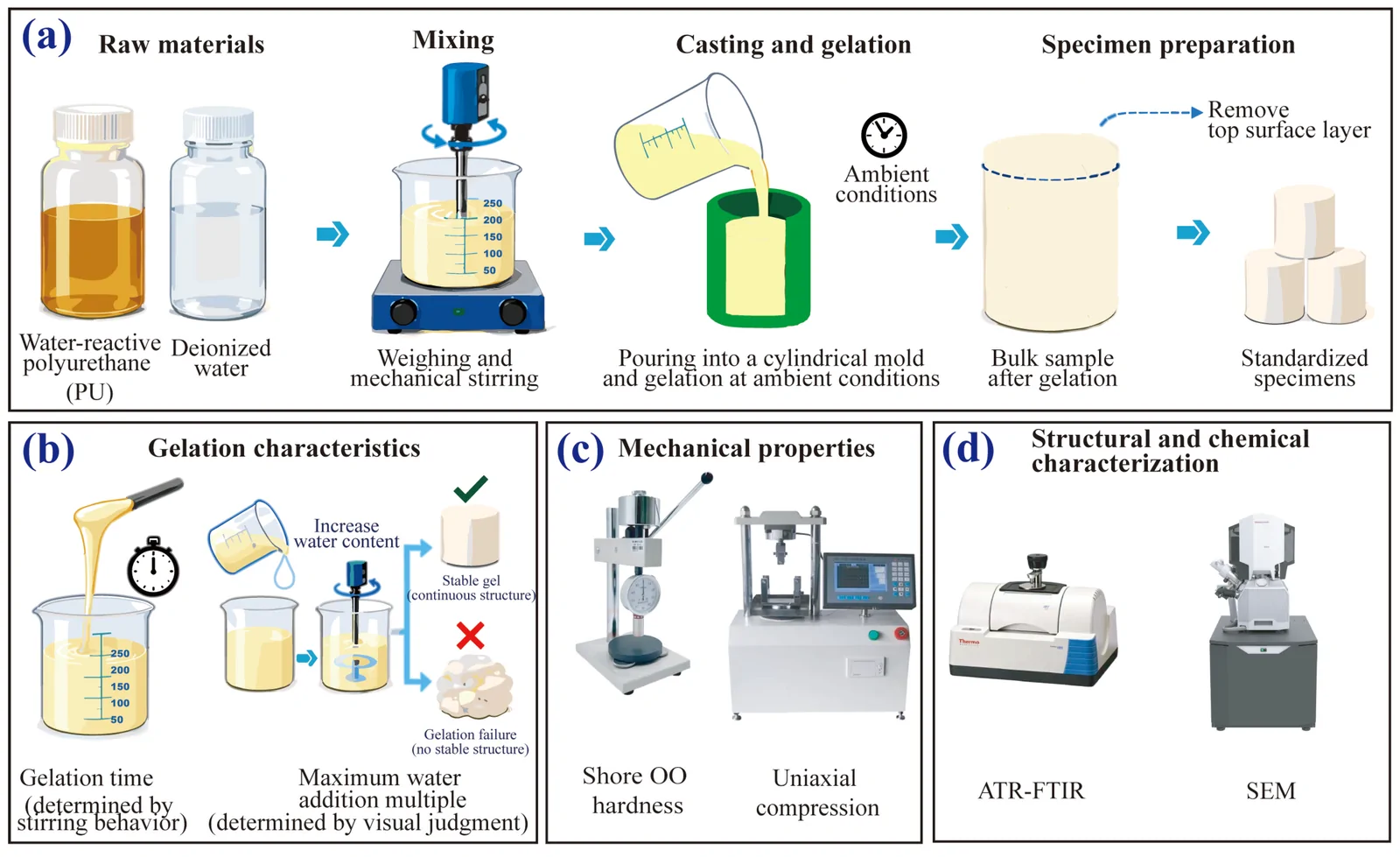
Specimen preparation and experimental scheme for water-reactive polyurethane: (**a**) specimen preparation; (**b**) gelation characterization; (**c**) mechanical testing; and (**d**) structural and chemical characterization.

Six groups of specimens were prepared with polyurethane-to-water volume ratios (PU:water, *v*/*v*) of 1:1, 1:2, 1:5, 1:10, 1:15, and 1:20. For specimen preparation, a predetermined volume of polyurethane was first placed in a container, followed by the addition of the corresponding volume of deionized water. The mixture was immediately stirred using an electric stirrer for 10 s to ensure adequate mixing of the two phases. Once a homogeneous mixture was obtained, stirring was stopped and the mixture was promptly cast. Prior to casting, the inner surfaces of the cylindrical silicone molds were uniformly coated with a polyurethane-compatible release agent. The mixed slurry was then immediately poured into cylindrical silicone molds with a diameter of 50 mm and a height of 50 mm and allowed to react at room temperature until stable consolidated specimens were formed. After consolidation, the specimens were demolded, and their uneven top surfaces were trimmed using a sharp utility knife to obtain flat surfaces for subsequent tests. After initial gel formation under ambient laboratory conditions, specimens intended for mechanical testing were placed in the same standard curing chamber overnight, following a consistent preparation and curing procedure across the tested PU:water ratios. The specimen preparation procedure and subsequent testing and characterization scheme are illustrated in Figure 1.

### 2.3. Determination of Gelation Time

The gelation time was measured with reference to JC/T 2041–2020 Polyurethane Grouting Materials [27] and ASTM D7487–24 Standard Practice for Polyurethane Raw Materials: Polyurethane Foam Cup Test [28], with the onset of stringing taken as the gelation endpoint. The components were mixed following the procedure described in Section 2.2, and timing was initiated upon contact between the deionized water and polyurethane. The state of the mixture was continuously observed, and timing was stopped when pronounced and continuous stringing was observed upon lifting the stirring rod; the elapsed time was recorded as the gelation time.

### 2.4. Determination of the Critical Water-to-Polyurethane Ratio for Gel Formation

The critical water-to-polyurethane ratio for gel formation was determined with reference to the water absorption capacity/amount tests specified in JC/T 2041–2020 [27] and DL/T 5785–2019 [29], with modifications based on the volumetric mix design adopted in this study. The water-addition multiple was defined as the ratio of the volume of added deionized water to the initial volume of polyurethane (Vwater/VPU). The PU:water volume ratio was further refined within the critical range identified in the preliminary tests, and stable gel formation was assessed by continuous macroscopic observation and a container-tilting check. A reacted mixture was classified as a stable gel when it formed a coherent, jelly-like body that retained its integrity and showed no visible flow when the container was tilted; mixtures that remained liquid and flowed upon tilting were classified as failing to form a stable gel. This qualitative endpoint was distinguished from the onset of stringing used to determine gelation time in Section 2.3.

### 2.5. Mechanical Property Tests

#### 2.5.1. Shore OO Hardness Test

Shore OO hardness was measured with reference to ASTM D2240-15(2021) [30] using an LX-OO durometer (Yueqing Handpi Instruments Co., Ltd., Yueqing, Zhejiang, China) equipped with a test stand. For each gel-forming PU:water ratio, one independently prepared specimen was used for hardness testing. Five measurements were taken at different surface locations on that specimen, and their average was reported as the Shore OO hardness. The dispersion among these five measurements describes within-specimen spatial variability, not between-specimen reproducibility.

#### 2.5.2. Compression Test

A single, consistent compression-testing procedure was adopted for all tested PU:water ratios. JC/T 2041–2020 [27] and DL/T 5785–2019 [29] were consulted as references for testing polyurethane and chemical grouting materials, while GB/T 1041–2008 [31] was consulted for general compression-testing principles. Cylindrical specimens with a diameter of 50 mm and a height of 50 mm were subjected to uniaxial compression using a YDW-300/10 microcomputer-controlled compression testing machine (Jinan Hengle Xingke Instruments Co., Ltd., Jinan, Shandong, China). The testing machine was equipped with a 10 kN load cell. Three independently prepared specimens were tested for each of the five PU:water ratios (1:1, 1:2, 1:5, 1:10, and 1:15). For each compression metric, the variability among independently prepared specimens was assessed separately from the five-location hardness measurements. The tests were performed under displacement control at a loading rate of 2 mm/min. During loading, force and displacement were continuously recorded, and the corresponding compressive stress–strain curves were calculated from the measured data. Because the compressive stress–strain curves were nonlinear, the compressive modulus was characterized by the secant modulus at 10% engineering strain (E_10_), calculated as E_10_ = σ_10_/0.10, where σ_10_ is the compressive stress at 10% strain and 0.10 is the corresponding dimensionless strain. This definition was applied consistently to all investigated PU:water ratios.

### 2.6. Scanning Electron Microscopy

To examine the microstructure of polyurethane specimens prepared at different water addition ratios, samples were taken from the central region of the cylindrical specimens and dried in a forced-air oven at 40 °C for 24 h. The dried specimens were sectioned using a sharp blade, and the newly exposed surfaces were sputter-coated with gold prior to observation using a scanning electron microscope (TESCAN MIRA LMS, Brno, Czech Republic). SEM images obtained at consistent magnifications were used to qualitatively compare local pore morphology, structural uniformity, and surface characteristics of specimens prepared at different water addition ratios. Some specimens with higher water addition exhibited partial pore deformation or collapse after oven drying; accordingly, the present two-dimensional images were not used to infer representative quantitative pore-size distributions.

### 2.7. Attenuated Total Reflectance Fourier Transform Infrared Spectroscopy

Attenuated total reflectance Fourier transform infrared spectroscopy (ATR-FTIR) was used to characterize the chemical structures of the PU prepolymer and the consolidated specimens prepared at PU:water volume ratios of 1:1, 1:2, 1:5, 1:10, and 1:15. Measurements were performed using a Nicolet iS5 FT-IR spectrometer equipped with an iD7 single-bounce diamond ATR accessory (Thermo Fisher Scientific, Madison, WI, USA). The consolidated specimens were dried under identical conditions prior to measurement, and a flat specimen surface was brought into close contact with the ATR crystal. The liquid PU prepolymer was measured directly using the ATR accessory. Spectra were recorded in absorbance mode over the wavenumber range of 4000–600 cm^−1^ at a spectral resolution of 4 cm^−1^, with 32 scans collected for each sample. The resulting spectra were used to compare the characteristic absorption bands associated with isocyanate, carbonyl/amide, N–H/O–H, and polyether groups.

### 2.8. Molecular Dynamics Simulation

Non-reactive all-atom molecular dynamics (MD) simulations were performed to characterize the pre-reaction molecular environment of PU–water systems with different water contents. An idealized MDI-capped, NCO-terminated polyether oligomer containing 157 atoms and two terminal isocyanate groups was adopted as a representative prepolymer model, and each simulation system contained 24 identical prepolymer chains. The model retained characteristic isocyanate, urethane-carbonyl, and polyether-oxygen sites relevant to PU–water interactions, but was not intended to reproduce the undisclosed chemical composition or molecular-weight distribution of commercial YH-201. The PU molecules were described using the General Amber Force Field version 2 (GAFF2) [32], with atomic partial charges assigned using the AM1-BCC scheme [33], while water was represented by the TIP3P model [34]. The nominal PU:water volume ratios of 1:1, 1:2, 1:5, 1:10, 1:15, and 1:20 were converted into molecular compositions using reference densities of 1.1487 g cm^−3^ for the neat prepolymer model and 0.997 g cm^−3^ for water, corresponding to 1276, 2551, 6378, 12,757, 19,135, and 25,514 water molecules, respectively. The prepolymer density used for this mapping was obtained from an NPT simulation of the neat molecular model rather than from experimental measurement of the commercial grout. Initial PU–water cells were constructed at a total mass density of 0.35 g cm^−3^, and periodic boundary conditions were applied in all three directions. The fixed-topology, non-reactive model was used to examine water localization and non-covalent contacts in an idealized uncured PU–water environment; it was not designed to simulate the water-curing reaction. Chemical conversion of –NCO groups, CO_2_ evolution, network formation, and curing kinetics were therefore outside its scope.

All simulations were performed using LAMMPS [35]. Short-range van der Waals and electrostatic interactions were evaluated using a cutoff distance of 9.0 Å, while long-range electrostatic interactions were treated using the particle–particle particle–mesh method [36] with a relative accuracy of 1 × 10^−4^. Each system was first subjected to conjugate-gradient energy minimization for up to 5000 iterations. This was followed by 50 ps of NVT simulation at 450 K, 200 ps of temperature-controlled isotropic compression at 450 K, 250 ps of NPT simulation at 450 K and 1 atm, and 200 ps of NPT cooling from 450 to 298.15 K at 1 atm. The systems were then conditioned under NPT conditions at 298.15 K and 1 atm for at least 800 ps; the 1:1 system was additionally extended by 1.0 ns. Nosé–Hoover temperature and isotropic pressure control were used, with a temperature damping time of 100 fs and pressure damping times of 5000 fs during the high-temperature NPT and cooling stages and 2000 fs during room-temperature NPT. Before production, each simulation cell was rescaled to the mean volume obtained over the final 100 ps of its NPT trajectory, followed by 50 ps of NVT settling at 298.15 K. A timestep of 0.5 fs was used through the cooling stage and 1.0 fs thereafter. Water O–H bonds and H–O–H angles were constrained using the SHAKE algorithm [37]. Production trajectories of 2 ns were generated under NVT conditions at 298.15 K for all six systems, while the 1:10 and 1:20 systems were subsequently extended to 5 ns. Unless otherwise specified, comparisons among compositions were based on the common 1.0–2.0 ns production window. Equilibration stability was examined from the final 400 ps of the room-temperature NPT trajectories using temperature, density, and potential-energy criteria; all six PU–water compositions satisfied those checks. Each composition was represented by one completed production trajectory, and independent production-run replicates were not performed. Extended trajectories for the 1:10 and 1:20 systems were continuations of their original runs rather than independent repeats. The equilibration checks are summarized in Supplementary Figure S1 and Table S1.

The local spatial distribution and intermolecular interactions of water were analyzed using water-O density fields, radial distribution functions (RDFs), coordination statistics, and hydrogen-bond analysis. Water-O density fields were averaged over the 1.0–2.0 ns interval using 500 frames sampled at 2 ps intervals; a target grid spacing of approximately 1.5 Å, Gaussian smoothing with σ = 1.5 Å, and a common density threshold of 0.01671 Å^−3^ were used for visualization. RDFs were calculated between water O atoms and three representative PU sites: the central C atom of the terminal –NCO group, urethane carbonyl O atoms, and ether O atoms. RDF calculations used the same 1.0–2.0 ns interval, 500 frames, and a radial bin width of 0.05 Å. A Gaussian filter with σ = 0.10 Å was used only to assist identification of RDF features, whereas the plotted RDF curves and cumulative coordination numbers were obtained from the original unsmoothed pair counts. First-shell water coordination numbers around carbonyl and ether O sites were determined from the first minima of the corresponding RDFs. For the –NCO central C, the number of water O atoms within a fixed radius of 4.0 Å was used as a local-neighborhood descriptor. Hydrogen bonds were identified using a donor–acceptor distance of ≤3.5 Å and a D–H···A angle of ≥150°, based on 100 frames sampled at 10 ps intervals. PU–water hydrogen bonding was normalized as NPU–water HB/Nwater, whereas water–water hydrogen-bond participation was defined as 2Nwater–water HB/Nwater.

## 3. Results and Discussion

### 3.1. Gelation Behavior and Maximum Water-Addition Multiple for Stable Gel Formation

**Figure 2:**
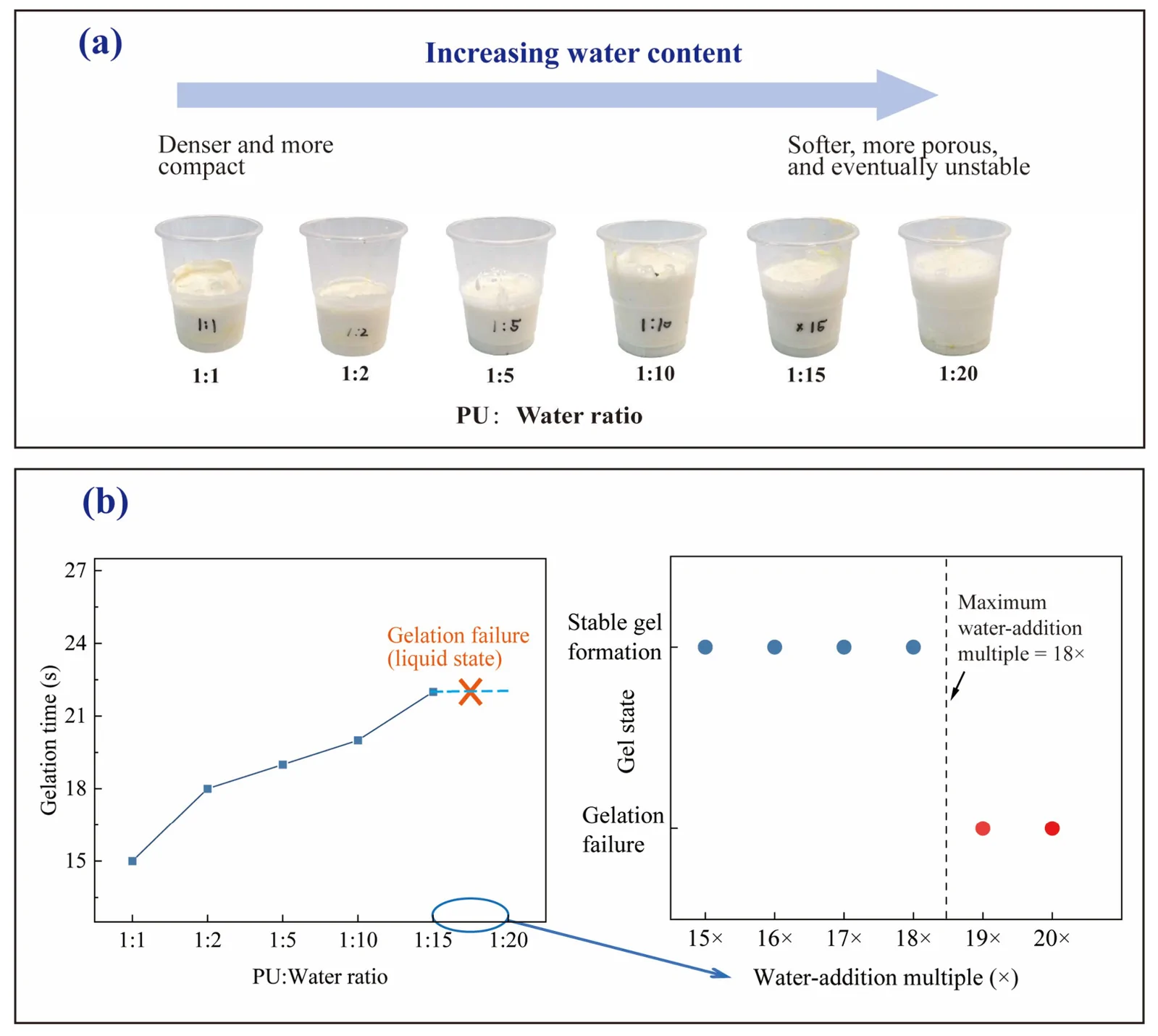
Gelation behavior of water-reactive polyurethane at different water addition ratios: (**a**) macroscopic gel states at different PU:water volume ratio; (**b**) gelation time and determination of the maximum water-addition multiple for stable gel formation.

The macroscopic gel states and gelation times of the water-reactive polyurethane at different water addition ratios are shown in Figure 2. As shown in Figure 2a, the morphology of the reacted material changed markedly with increasing water addition. At a PU:water volume ratio of 1:1, the consolidated material exhibited good integrity and a relatively compact appearance. As the water addition increased, the material gradually became softer and exhibited more pronounced porous features, although a complete and stable gel was still formed at PU:water = 1:15. When the water addition was further increased to 1:20, the system could no longer form a stable and integral gel, indicating gelation failure. Figure 2b shows that, for the specimens capable of stable gel formation, the gelation time generally increased with increasing water addition, from approximately 15 s at PU:water = 1:1 to approximately 22 s at PU:water = 1:15. These results indicate that increasing water addition progressively delayed the transition from a fluid state to a stable gel state and ultimately led to the loss of stable gel-forming ability at high water contents.

To further determine the upper limit of stable gel formation, additional mix ratios were tested within the high-water-addition range. As shown in Figure 2b, the mixtures at water-addition multiples of 15×–18× formed coherent, non-flowing gels under the macroscopic tilting criterion, whereas the 19× and 20× mixtures remained flowable and did not meet that criterion. Therefore, under the present experimental conditions, the maximum water-addition multiple allowing stable gel formation was determined to be 18×, corresponding to a PU volume ratio of 1:18. This result indicates that, although the material can accommodate a relatively large amount of added water while maintaining gel formation, its stable gel-forming capacity has a clear upper limit under the tested conditions. Once the water addition exceeds this critical range, the relative amount of polyurethane available to form a continuous consolidated structure may become insufficient, ultimately resulting in gelation failure.

### 3.2. Mechanical Properties

**Figure 3:**
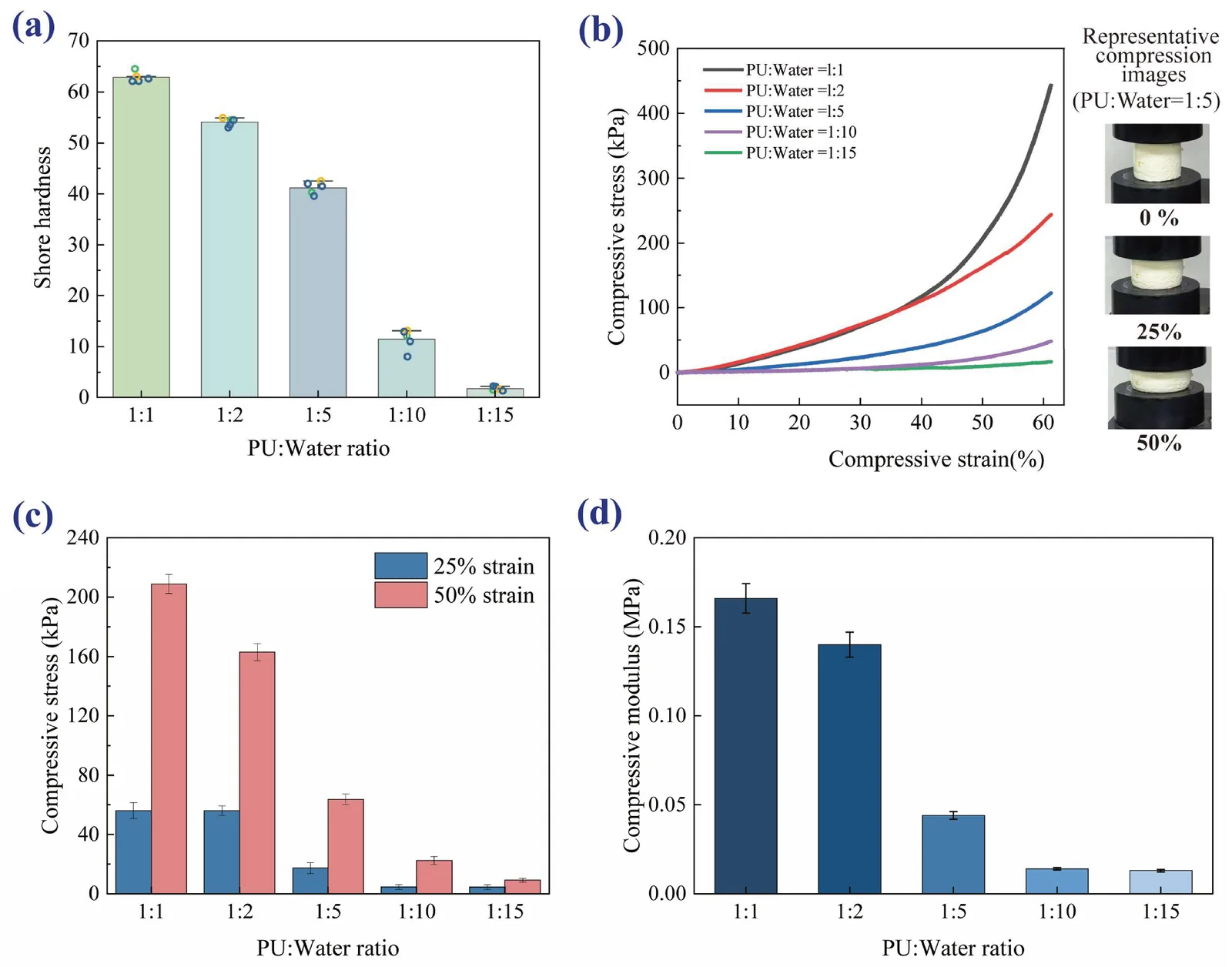
Mechanical properties of water-reactive polyurethane at different water addition ratios: (**a**) Shore OO hardness; (**b**) compressive deformation states and corresponding stress–strain curves; (**c**) compressive stresses at 25% and 50% strain; (**d**) compressive modulus at 10% strain.

The Shore OO hardness of the polyurethane specimens prepared at different water addition ratios is shown in Figure 3a. The five location measurements on each specimen yielded a within-specimen sample standard deviation ranging from approximately 0.39 to 2.08 Shore OO units across the investigated ratios; these values do not characterize between-specimen variability. The hardness decreased markedly with increasing water addition. At PU:water = 1:1, the Shore OO hardness was approximately 63. It decreased to approximately 54 and 41 at PU:water = 1:2 and 1:5, respectively, and further declined to approximately 11 and 2 at PU:water = 1:10 and 1:15. The decrease in hardness was relatively gradual at lower water additions but became much more pronounced as the water addition increased beyond PU:water = 1:5. These results indicate that increasing water content progressively changed the consolidated polyurethane from a relatively hard material to a much softer one, consistent with the macroscopic changes observed in Figure 2.

Figure 3b presents the deformation states of representative specimens at different compressive strains together with the compressive stress–strain curves obtained at different water addition ratios. The specimens maintained their overall integrity even under relatively large compressive deformation, exhibiting pronounced large-deformation behavior. For all groups, the compressive stress increased continuously with strain and exhibited a nonlinear response, although clear differences were observed among the different water addition ratios. The specimens at PU:water = 1:1 and 1:2 showed similar compressive responses at relatively low strains, whereas the difference between them became more pronounced as the strain increased. With further increases in water addition to PU:water = 1:5, 1:10, and 1:15, the stress–strain curves shifted markedly downward, indicating a substantial reduction in the resistance of the consolidated material to compressive deformation.

To further quantify the compressive behavior, the compressive stresses at 25% and 50% strain and the compressive modulus were extracted, as shown in Figure 3c,d. At 25% strain, the compressive stresses of the PU:water = 1:1 and 1:2 specimens were both approximately 55 kPa, whereas those of the 1:5, 1:10, and 1:15 specimens decreased to approximately 17, 4, and 4 kPa, respectively. At 50% strain, the corresponding compressive stresses were approximately 208, 163, 63, 22, and 8 kPa. The compressive modulus also showed an overall decreasing trend with increasing water addition, remaining relatively high for the 1:1 and 1:2 specimens but decreasing markedly at higher water additions. Overall, the Shore OO hardness and compression test results demonstrate that increasing water addition substantially reduces the hardness, stiffness, and compressive load-bearing capacity of the consolidated polyurethane, while increasing its deformability. Such reductions may diminish the resistance of a formed seal to local mechanical or hydraulic disturbance; however, water-sealing efficiency cannot be inferred directly from compression and hardness tests alone, because permeability, grout retention, interfacial contact, and flow conditions also govern field performance.

### 3.3. Microstructural Evolution at Different Water Addition Ratios

**Figure 4:**
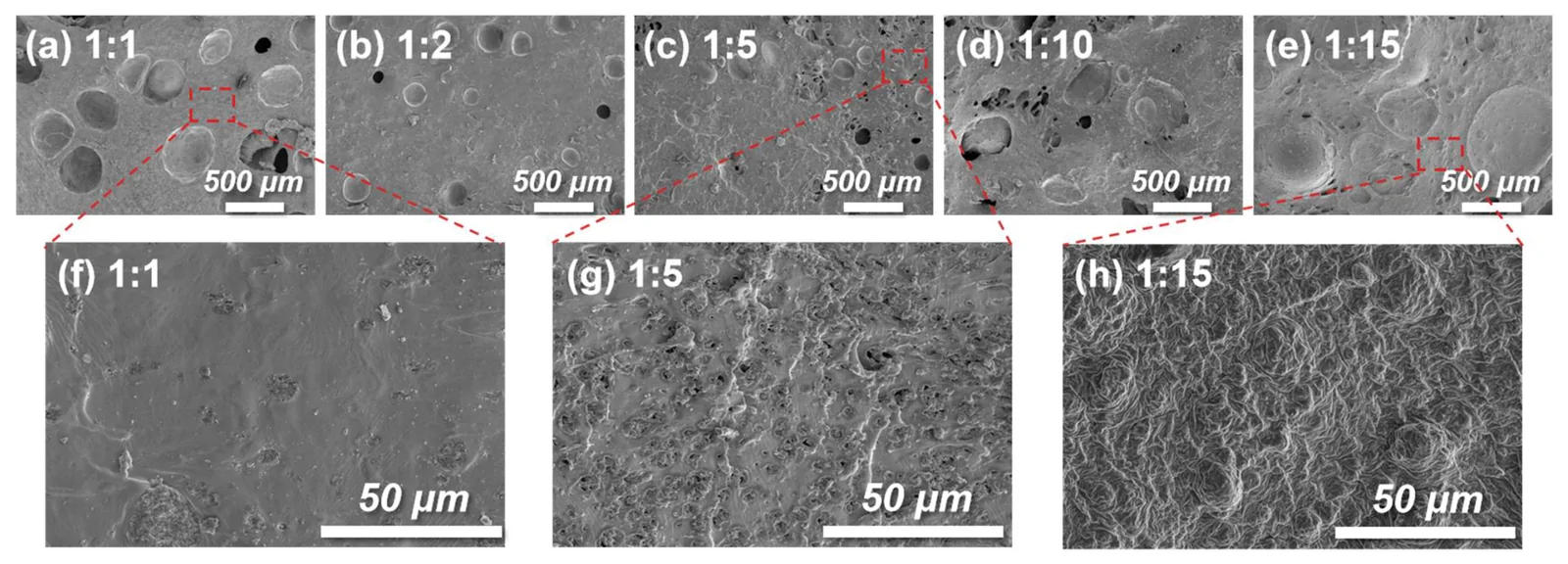
SEM images of water-reactive polyurethane prepared at different water addition ratios: (**a**–**e**) low-magnification images at PU:water ratios of 1:1, 1:2, 1:5, 1:10, and 1:15, respectively; (**f**–**h**) representative high-magnification images of the 1:1, 1:5, and 1:15 specimens, respectively.

SEM images of the polyurethane specimens prepared at different water addition ratios are shown in Figure 4. As shown in Figure 4a–e, all specimens exhibited a distinct porous structure, with pores that were predominantly circular or elliptical. However, clear differences in pore morphology, size distribution, and structural uniformity were observed as the water addition increased. At PU:water = 1:1 and 1:2, the pore contours were relatively regular and the surrounding matrix remained comparatively continuous. When the water addition increased to PU:water = 1:5 and 1:10, the coexistence of pores with different sizes became more pronounced, accompanied by an increased occurrence of irregularly shaped pores. At PU:water = 1:15, pore boundaries became less distinct and the local morphology appeared more irregular (Figure 4e). These qualitative observations suggest changes in the observed morphology and local structural uniformity with increasing water addition; they do not establish a quantitative pore-size distribution.

Figure 4f–h present representative high-magnification images of the PU:water = 1:1, 1:5, and 1:15 specimens. The local matrix of the 1:1 specimen appeared relatively smooth and continuous. At PU:water = 1:5, the surface became noticeably rougher, with more fine pores and irregular features. When the water addition increased to 1:15, the local surface exhibited pronounced wrinkling and partly collapsed-looking features (Figure 4h). Because the specimens were oven-dried before SEM examination, moisture-removal-induced deformation may have contributed to these features, particularly at high water addition; this possibility prevents an unambiguous interpretation of the original pore geometry. Similar effects of water content have been reported for water-blown polyurethane systems, in which increasing water addition altered cell growth, intercellular connectivity, and the morphology of the polymer skeleton [21]. These changes are closely associated with the foaming process induced by the reaction between water and isocyanate groups, during which variations in water content affect the interplay among gas generation, cell growth, and polymer curing, thereby modifying the final pore structure [13,18,20,21]. The observed local morphological changes are consistent with the substantial reduction in mechanical resistance shown in Figure 3, but the possible effects of drying mean that a direct quantitative relationship between original pore sizes and mechanical properties cannot be established from these images.

### 3.4. Chemical Structure at Different Water Addition Ratios

**Figure 5:**
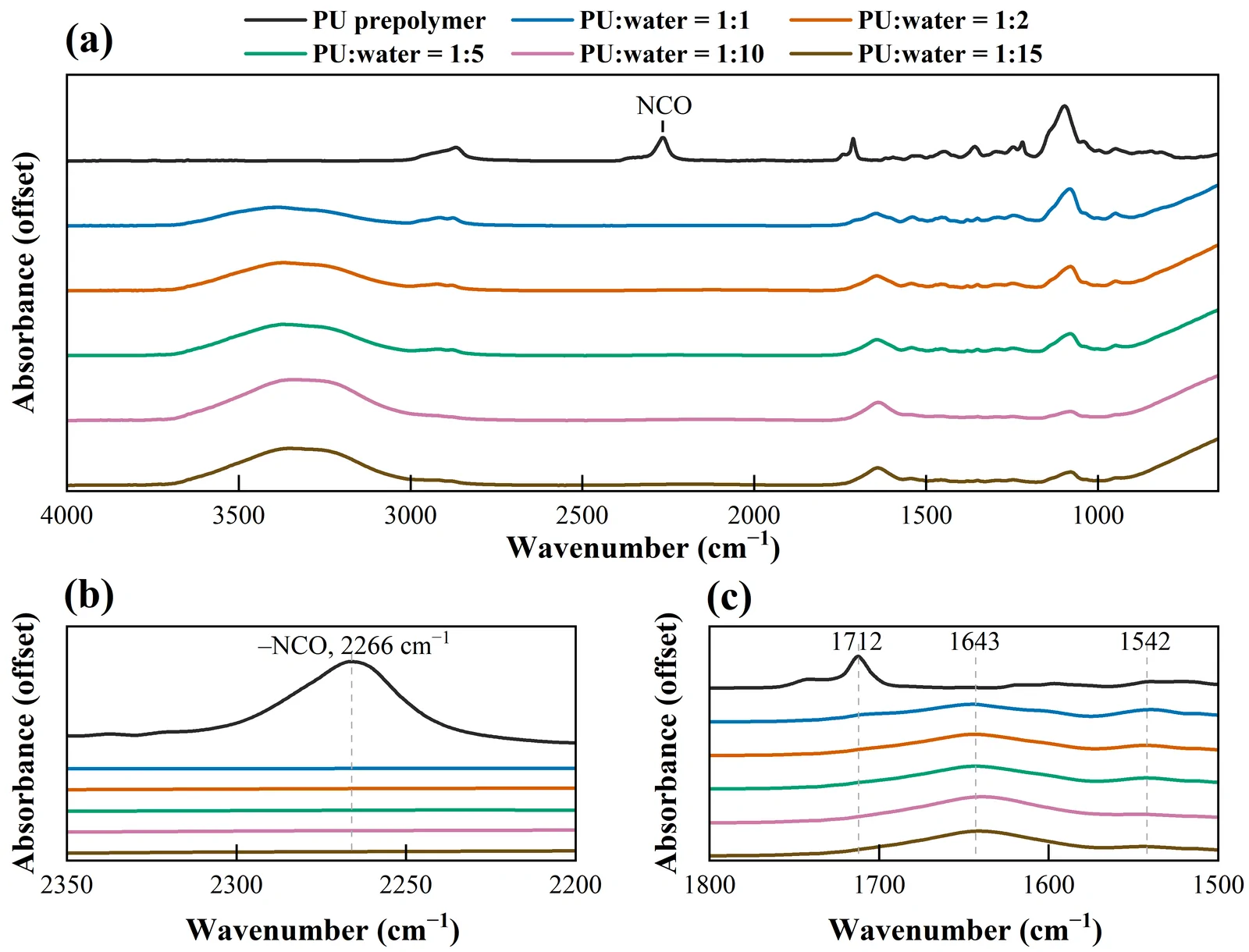
ATR-FTIR spectra of the PU prepolymer and consolidated specimens prepared at different PU:water ratios: (**a**) full spectra; (**b**) enlarged isocyanate region; and (**c**) enlarged carbonyl/amide region.

The ATR-FTIR spectra of the PU prepolymer and the consolidated specimens prepared at different PU:water ratios are shown in Figure 5a. The cured specimens exhibit broadly similar spectral profiles, with the principal absorption bands remaining at comparable wavenumbers over the investigated range. The broad band in the 3300–3400 cm^−1^ region is primarily associated with N–H stretching, with possible contributions from hydrogen-bonded O–H groups, while the band near 2920 cm^−1^ originates from aliphatic C–H stretching. In the fingerprint region, the bands around 1240 and 1080 cm^−1^ are mainly associated with C–N/C–O vibrations and C–O–C stretching of the polyether segments, respectively [38]. These common spectral features indicate that the cured specimens retain a similar overall PU-based chemical framework. More distinct differences are observed in the isocyanate and carbonyl/amide regions, as discussed below.

As shown in Figure 5b, the PU prepolymer exhibits a pronounced absorption band at 2266 cm^−1^, corresponding to the stretching vibration of free isocyanate (–NCO) groups [39]. In contrast, no distinct –NCO band is observed in the consolidated specimens prepared at PU:water ratios from 1:1 to 1:15, indicating substantial consumption of free isocyanate groups during curing. In water-reactive polyurethane systems, –NCO groups react with water through an unstable carbamic acid intermediate, which subsequently decomposes to generate a primary amine and CO_2_; the resulting amine can further react with isocyanate groups to form urea linkages [13,14]. Accordingly, the disappearance of the 2266 cm^−1^ band is consistent with extensive conversion of free isocyanate during water-triggered curing and with the associated CO_2_-generating foaming mechanism.

Figure 5c further highlights the carbonyl and amide regions, where the structural changes associated with the isocyanate–water reaction are more clearly resolved. The PU prepolymer exhibits a distinct carbonyl band near 1712 cm^−1^, which is consistent with hydrogen-bonded urethane C=O groups. After reaction with water, the consolidated specimens show a broader carbonyl envelope centered predominantly around 1643 cm^−1^. In polyurethane–urea systems, carbonyl bands in the vicinity of 1640–1670 cm^−1^ are commonly associated with hydrogen-bonded urea structures, whereas hydrogen-bonded urethane carbonyls generally occur near 1710 cm^−1^. The band near 1542 cm^−1^ is assigned to the amide II vibration, arising mainly from N–H bending coupled with C–N stretching [40]. The positions of these characteristic bands remain broadly similar among the cured specimens from PU:water = 1:1 to 1:15, although differences in the carbonyl-band profiles are evident. These spectral variations are consistent with changes in the local urethane/urea carbonyl environments and hydrogen-bonding interactions rather than the formation of a fundamentally different chemical framework. Because the spectra were vertically offset and no carbonyl-band deconvolution or internal-standard normalization was performed, differences in band intensity were not interpreted quantitatively.

### 3.5. Molecular-Level Structure and Interactions in Pre-Reaction PU–Water Systems

**Figure 6:**
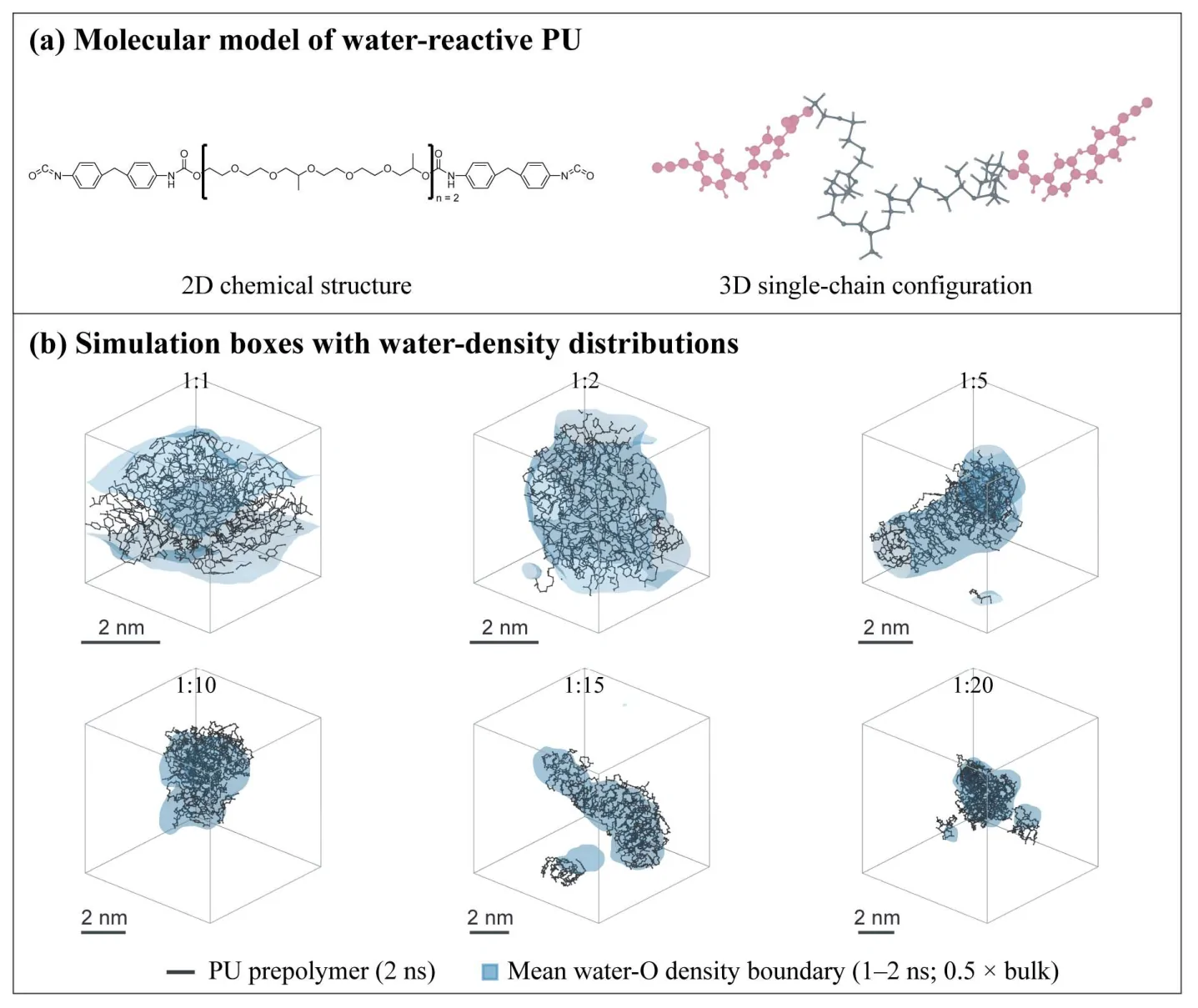
Molecular model and representative configurations of PU–water systems: (**a**) molecular model of the NCO-terminated PU prepolymer; (**b**) representative PU configurations and time-averaged water-O density boundaries at different PU:water ratios.

To provide molecular-level insight into the initial contact between the water-reactive PU prepolymer and water prior to chemical reaction, non-reactive all-atom MD simulations were performed for systems with different water contents. Figure 6a presents the representative NCO-terminated PU prepolymer model used in the simulations, including its two-dimensional chemical structure and a three-dimensional single-chain configuration. Figure 6b shows representative PU configurations at 2 ns together with the corresponding water-O density boundaries averaged over the common 1–2 ns production window. Figure 6b provides a qualitative visualization of the different system compositions and the corresponding time-averaged spatial distributions of water O atoms. The density boundaries represent time-averaged water distributions rather than instantaneous hydration shells around the displayed 2 ns configurations. Because these visualizations provide primarily qualitative information on the pre-reaction molecular environment, site-specific radial distributions, neighboring-water statistics, and hydrogen-bond characteristics were further analyzed quantitatively.

**Figure 7:**
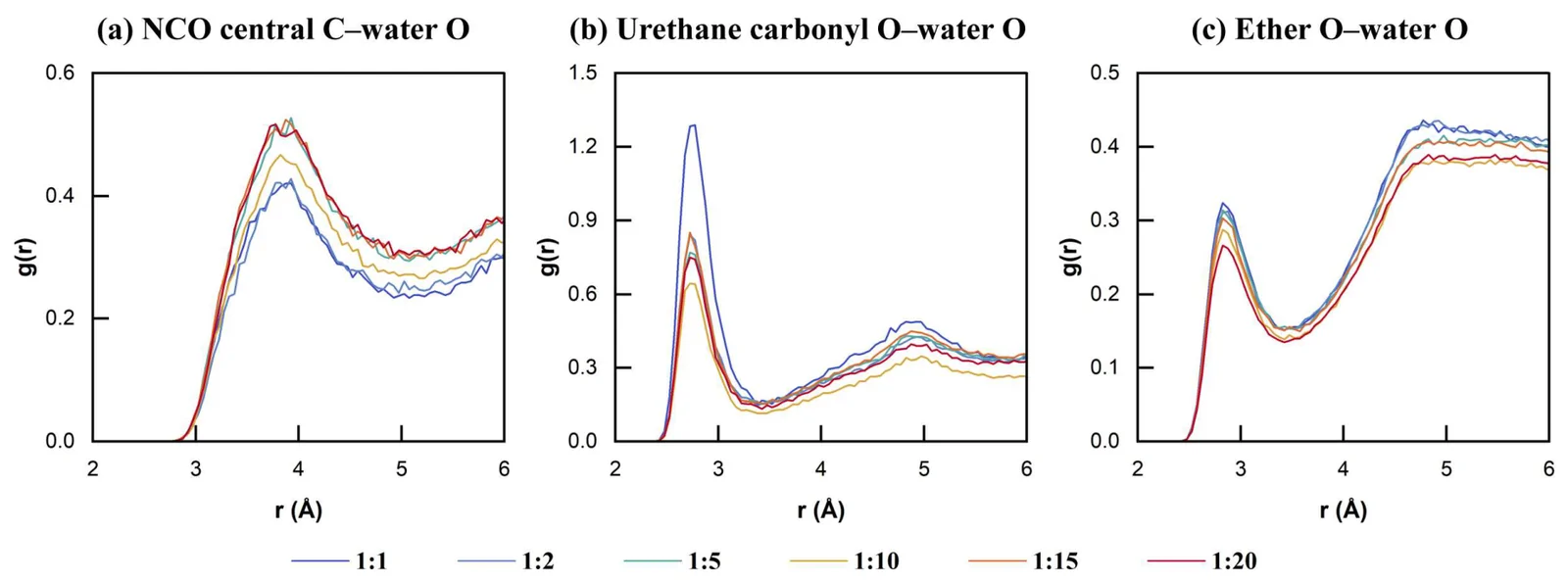
Radial distribution functions of water around representative PU sites: (**a**) NCO central C–water O; (**b**) urethane carbonyl O–water O; (**c**) ether O–water O.

**Figure 8:**
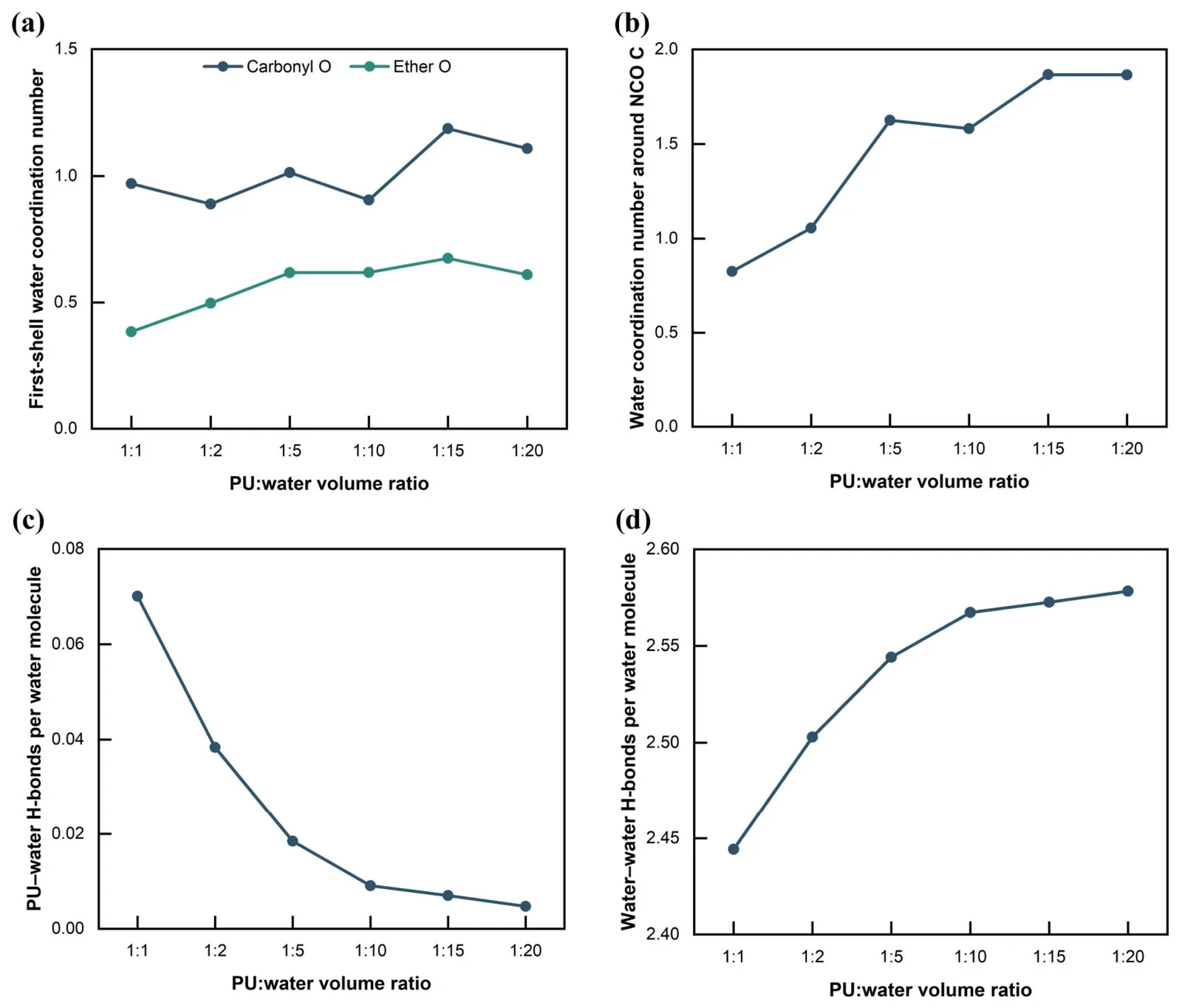
Local water coordination and hydrogen-bond statistics in PU–water systems: (**a**) first-shell water coordination numbers around carbonyl and ether O sites; (**b**) number of water O atoms within 4.0 Å of the NCO central C; (**c**) PU–water hydrogen bonds per water molecule; (**d**) water–water hydrogen-bond participation per water molecule.

Radial distribution functions (RDFs) between water O atoms and three representative PU sites were calculated over the common 1–2 ns production window, as shown in Figure 7. For the central C atom of the –NCO group, a broad radial maximum occurs at approximately 3.8–3.9 Å (Figure 7a). Its position remains nearly unchanged among the six compositions, whereas the peak magnitude varies non-monotonically with water content. The urethane carbonyl O–water O RDF exhibits a distinct first-shell radial feature near 2.8 Å (Figure 7b). The corresponding peak is highest at PU:water = 1:1 and lower at the other compositions, although the variation among the water-rich systems is non-monotonic. A first-shell radial feature near 2.9 Å is similarly observed for the ether O–water O pair (Figure 7c). Overall, the radial positions of the three site pairs remain broadly unchanged with composition, indicating that their characteristic short-range separations are relatively insensitive to water content within the analyzed window. Because the RDFs are normalized by the overall water number density of each system, differences in peak height reflect changes in relative radial correlations rather than absolute numbers of neighboring water molecules. The local water populations are therefore evaluated separately using the statistics shown in Figure 8.

Figure 8 further quantifies the local water environment and hydrogen-bond statistics within the same 1–2 ns production window. The first-shell water coordination numbers around the carbonyl and ether O sites, determined from the corresponding RDF minima, exhibit composition-dependent but non-monotonic variations (Figure 8a). For the central C atom of the –NCO group, the fixed 4.0 Å local-neighborhood descriptor defined in Section 2.8 generally increases from approximately 0.83 at PU:water = 1:1 to approximately 1.87 at 1:15–1:20, with a small decrease at 1:10 (Figure 8b). The average number of PU–water hydrogen bonds per water molecule decreases markedly from approximately 0.070 to 0.0047 as the water content increases (Figure 8c). Because the number of PU hydrogen-bonding sites remains fixed while the water population increases substantially, this decrease contains an inherent dilution contribution and should not be interpreted as evidence of a weakening of the intrinsic PU–water interaction strength. In contrast, the water–water hydrogen-bond participation per water molecule, defined as twice the number of water–water hydrogen bonds divided by the number of water molecules, increases modestly from approximately 2.44 to 2.58, corresponding to an increase of about 5.5% (Figure 8d). Thus, the present statistics indicate a pronounced decrease in the normalized participation of individual water molecules in PU–water hydrogen bonding together with only a modest increase in water–water hydrogen-bond participation; they do not, by themselves, demonstrate the formation of distinct water clusters or a continuous hydrogen-bond network.

Taken together, the non-reactive MD simulations show that changing the PU:water ratio is accompanied by measurable changes in the local molecular environment before chemical reaction. All composition comparisons presented above are based on the common 1–2 ns production window. Comparison with the 4–5 ns window for the 1:10 and 1:20 systems revealed appreciable time-window sensitivity in the number of water O atoms within 4.0 Å of the NCO central C (approximately 1.58 to 1.20 and 1.87 to 1.41, respectively, corresponding to decreases of about 24%); accordingly, the present MD results are interpreted as window-specific structural observations rather than fully converged equilibrium values. These later windows belong to continuations of the same trajectories and cannot be treated as independent replicates. Their RDF time-window comparison and NCO-neighborhood statistics are provided in Supplementary Figure S2 and Table S2. Within this limitation, the simulations provide molecular-scale context for the composition-dependent behavior observed experimentally by showing that water addition affects the short-range distribution and hydrogen-bond participation of water in the initial PU–water system. However, the present fixed-topology simulations do not model isocyanate conversion, CO_2_ generation, pore growth, gelation kinetics, or formation of the cured polymer network. Accordingly, the MD results are used here as molecular-level background for interpreting the effects of water addition rather than as direct evidence for the mechanisms governing gelation, foaming, or mechanical-property evolution.

## 4. Conclusions

The effects of water addition on the gelation behavior, mechanical properties, microstructure, chemical structure, and pre-reaction molecular environment of a water-reactive PU grouting material were investigated. The main conclusions are as follows:

1. Increasing water addition delayed gel formation and eventually resulted in the loss of stable gel-forming ability. The gelation time increased from approximately 15 s at PU:water = 1:1 to approximately 22 s at 1:15. Stable gels could be formed up to a water-addition multiple of 18× (PU:water = 1:18, *v*/*v*), whereas gel formation failed at 19× and 20×, demonstrating a distinct upper limit of stable gel formation under the present experimental conditions.

2. Water addition markedly reduced the mechanical resistance of the consolidated PU. The Shore OO hardness decreased from approximately 63 at PU:water = 1:1 to approximately 2 at 1:15, while the compressive stress at 50% strain decreased from approximately 208 to 8 kPa. The compressive modulus showed a similar overall decline, indicating that increasing water content produced a progressively softer and more deformable consolidated material.

3. Increasing water addition altered the pore morphology and structural uniformity rather than simply causing a monotonic increase in pore size. SEM observations revealed increasingly irregular, heterogeneous, and locally loose structures at high water contents. ATR-FTIR spectra showed the disappearance of the free –NCO band at 2266 cm^−1^ after curing and corresponding changes in the carbonyl/amide region, consistent with extensive isocyanate consumption and the development of polyurethane–urea structures, while the cured specimens retained a broadly similar overall chemical framework.

4. Non-reactive MD simulations further indicated composition-dependent changes in the pre-reaction PU–water environment. Increasing water content altered the local distribution of water around representative PU sites, markedly reduced PU–water hydrogen-bond participation per water molecule, and produced only a modest increase in water–water hydrogen-bond participation. These simulations provide molecular-scale context for the experimental observations but, because of their fixed-topology and time-sensitive nature, should not be interpreted as direct evidence for the mechanisms of chemical reaction, foaming, or gelation.

Several limitations should be noted. The five Shore OO hardness readings for each ratio were collected from one specimen and therefore quantify local measurement variability rather than between-specimen repeatability. SEM observations were qualitative and may have been affected by drying-induced pore deformation at high water addition. In addition, the molecular simulations employed a representative, fixed-topology prepolymer model, and some local structural descriptors remained time-window-dependent without independent production-run replication. Finally, mechanical tests alone do not quantify permeability, washout resistance, or actual water-sealing effectiveness. Future studies should address these issues through replicated specimen-level measurements, drying-minimized pore characterization, longer and independently repeated molecular simulations where appropriate, and direct hydraulic sealing tests.

Overall, these results demonstrate the effects of water addition on the gel-forming capacity, mechanical response, and multiscale structural characteristics of the investigated water-reactive PU, providing a basis for evaluating its water tolerance and controlling the PU–water proportion in water-rich grouting applications. Future research may also explore data-driven approaches for predicting grout performance under varying water-addition conditions, drawing methodological inspiration from hybrid forecasting frameworks developed for other engineering applications [41].

## Data Availability

The raw data supporting the conclusions of this article will be made available by the authors on request.

## Supplementary Materials

The following supporting information can be downloaded at: https://www.mdpi.com/article/10.3390/polym18212731/s1, Figure S1: Thermodynamic equilibration assessment of PU–water systems; Figure S2: Time-window sensitivity of molecular structural descriptors; Table S1: Molecular model and simulation parameters; Table S2: Equilibration and time-window assessment.
