# Supplementary material for: Effect of Water Addition on Gel-Forming Capacity and Structure–Property Evolution of Water-Reactive Polyurethane Grout
Source: Polymers (Basel). 2026 Nov 3;18(21):2731. doi: 10.3390/polym18212731 (PMC13766194; doi:10.3390/polym18212731)
Supplement: Supplementary file 1 [file polymers-18-02731-s001.zip › polymers-4657672-Supplementary_Information.pdf]

Supplementary Information

Effect of Water Addition on Gel-Forming Capacity and Structure–Property Evolution of Water-Reactive Polyurethane Grout

Bowen Qi, Xinhua Chen, Langtian Qin, and Feng Huang

School of Engineering and Technology, China University of Geosciences (Beijing), Beijing 100083, China

The molecular dynamics (MD) model describes an uncured, representative polyurethane (PU) prepolymer–water mixture. It does not reproduce the complete proprietary formulation of YH-201. The fixed-topology simulations characterize pre-reaction molecular environments and do not simulate chemical conversion, gas evolution, crosslinking, or gel formation.

Table S1. Molecular model and simulation parameters.

Parameter	Specification
Representative PU model	MDI-capped, NCO-terminated polyether oligomer; $C_{58}H_{78}N_4O_{17}$; 1103.273 g mol^{-1}
PU chains	24 identical chains per system; 157 atoms and two NCO groups per chain
Force field and water	GAFF2, AM1-BCC partial charges; rigid TIP3P water with SHAKE
Nominal PU:water ratios (v/v)	1:1, 1:2, 1:5, 1:10, 1:15, 1:20
Water molecules, respectively	1276, 2551, 6378, 12,757, 19,135, 25,514
Density for composition mapping	1.1487 g cm^{-3} for the neat model; 0.997 g cm^{-3} for water
Equilibration	450 K relaxation and compression; NPT cooling to 298.15 K; 298.15 K NPT for 800 ps, with 1000 ps additional NPT for 1:1
Production	50 ps NVT settling, then 2 ns NVT at 298.15 K; 1:10 and 1:20 continued to 5 ns
Analysis windows	1–2 ns for all compositions; 4–5 ns for the two extended trajectories
Sampling	RDF: every 2 ps; hydrogen bonds and local time series: every 10 ps

Table S2. Equilibration and time-window assessment.

Final 400 ps of the 298.15 K NPT stage. Density and energy differences are second-half minus first-half means. The temperature and density are window means.

**PU:water**	**298 K NPT (ps)**	**T (K)**	**Density (g cm^{-3})**	**Δρ halves (%)**	**ρ drift (%)**	**ΔPE halves (kcal mol^{-1} atom^{-1})**
1:1	1800	298.26	1.07766	+0.035	+0.109	-0.0018
1:2	800	298.17	1.04636	+0.045	+0.036	+0.0014
1:5	800	298.16	1.01547	+0.084	+0.125	-0.0022
1:10	800	298.11	1.00193	+0.009	+0.021	-0.0001
1:15	800	298.24	0.99667	+0.005	+0.018	-0.0005
1:20	800	298.09	0.99418	-0.013	-0.012	+0.0003

Screening limits were $|T - 298.15| \le 1.5$ K, $|\Delta\rho$ halves$| \le 0.15\%$, $|\rho$ drift$| \le 0.25\%$, and $|\Delta PE$ halves$| \le 0.005$ kcal mol^{-1} atom^{-1}. All reported values meet these limits. The recorded density rule also considered block uncertainty when flagging threshold exceedances. The fixed density in the subsequent NVT production stage is not an independent density-convergence test.

Local descriptors in the early and late windows

**PU:water**	**Window (ns)**	**NCO-C water/C**	**NCO-O water/O**	**PU–water HB/chain**	**Energy/chain (kcal mol^{-1})**
1:10	1–2	1.581	1.165	4.826	-81.54
1:10	4–5	1.203	0.880	4.311	-71.99
1:20	1–2	1.865	1.383	5.010	-88.42
1:20	4–5	1.413	1.047	4.825	-81.02

NCO-C and NCO-O values are the mean numbers of water O atoms within 4.0 Å of the NCO central C and 3.5 Å of the NCO O, respectively. NCO-C values use 2 ps RDF sampling; NCO-O, hydrogen-bond and energy values use 10 ps sampling. Hydrogen bonds use donor–acceptor distance ≤ 3.5 Å and donor–H–acceptor angle ≥ 150°. Energy is the PU–water group/group pair plus reciprocal-space contribution per chain. Later windows are continuations, not independent replicates; one completed production trajectory was available per composition.

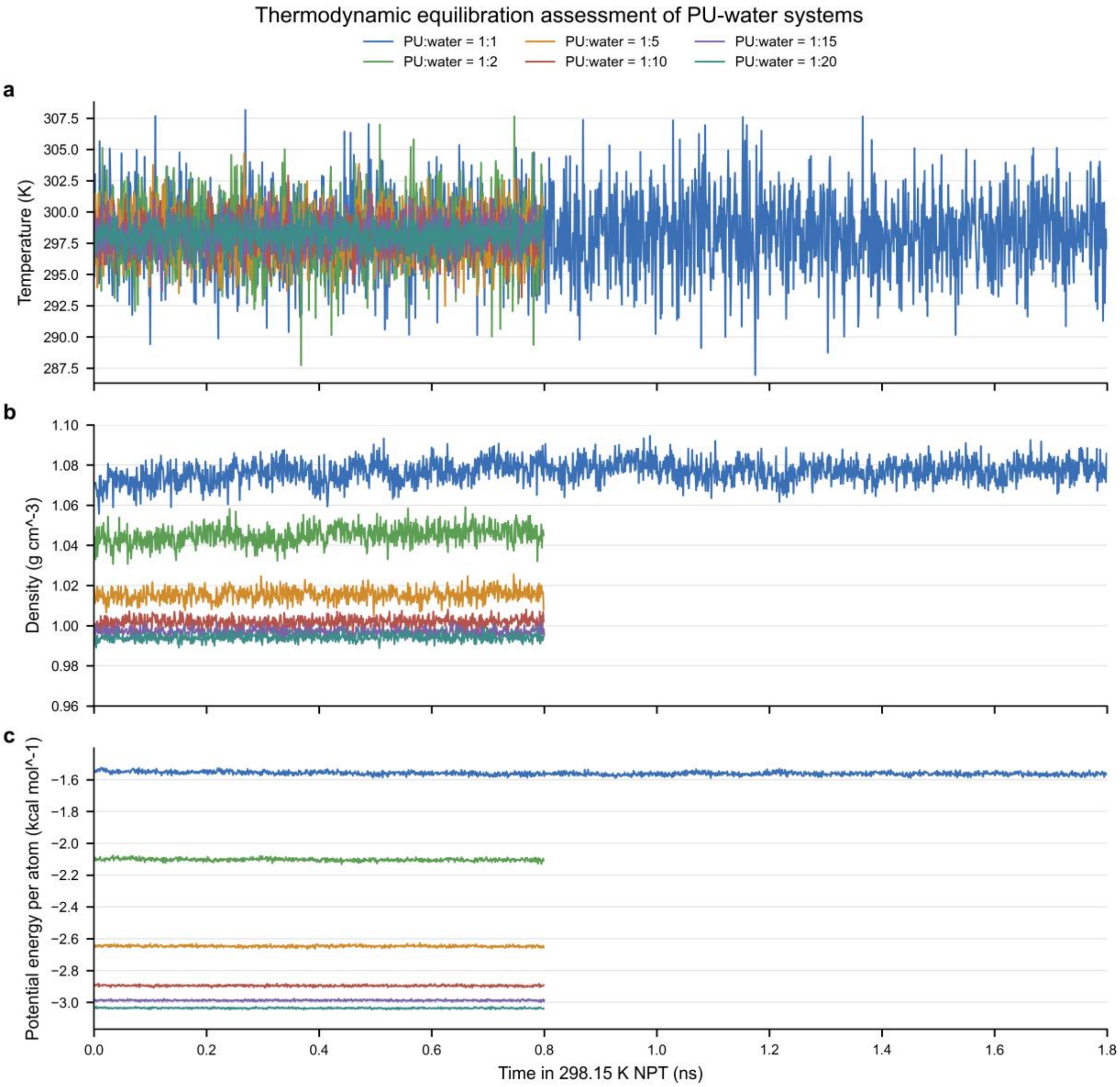

Figure S1. Thermodynamic equilibration assessment of PU–water systems. Temperature, density and potential energy per atom are shown for the final room-temperature NPT segments of the six compositions. The 1:1 trace covers 1800 ps and the other traces cover 800 ps. The final 400 ps was used for the screening diagnostics in Table S2. Each curve represents one simulation; stability of these thermodynamic quantities does not establish convergence of local structure.

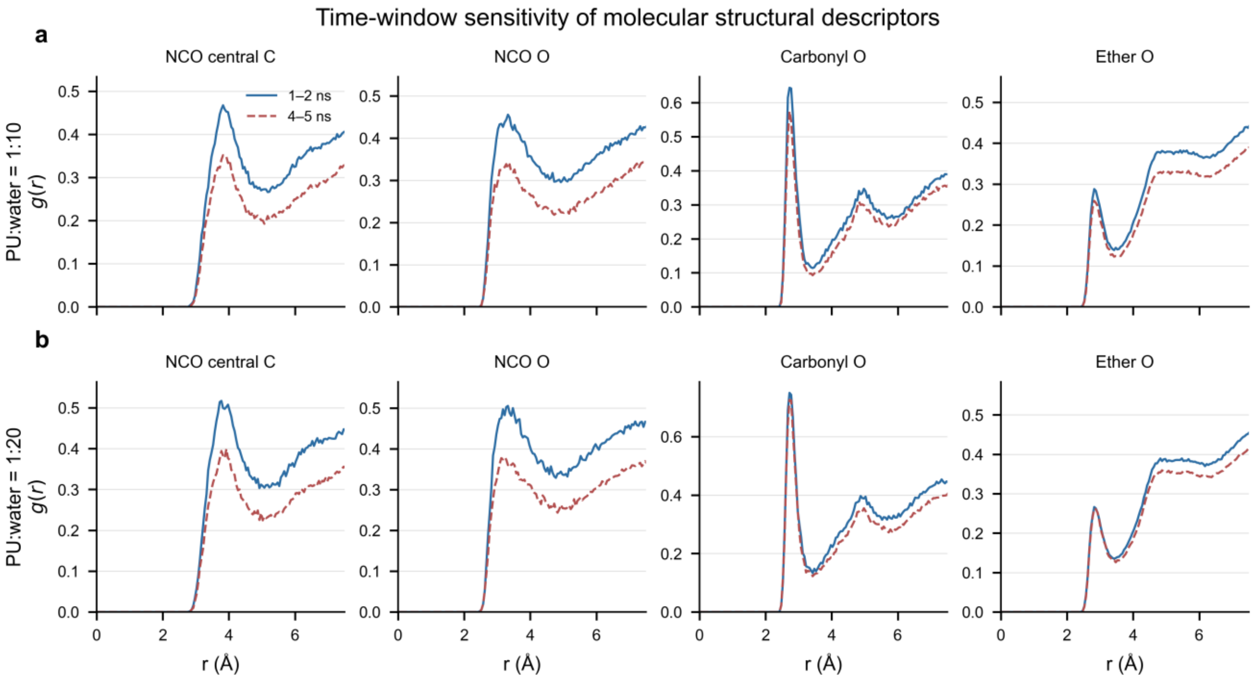

Figure S2. Time-window sensitivity of molecular structural descriptors. Radial distribution functions between water O and the indicated PU sites are shown for (a) PU:water = 1:10 and (b) 1:20 in the 1–2 ns and 4–5 ns production windows. The latter windows continue the same trajectories. Water O counts within 4.0 Å of the NCO central C decreased from 1.581 to 1.203 for 1:10 and from 1.865 to 1.413 for 1:20 (Table S2). These differences indicate time-window sensitivity; full convergence of local descriptors was not established.

RDFs were obtained with a 0.05 Å bin width and normalized by the overall water oxygen number density in each simulation cell. Fixed-radius neighborhood counts describe local water proximity and are not measures of reaction rate.
